# A Novel Automated Calculation of Basal Cistern Effacement Status on Computed Tomographic Imaging in Traumatic Brain Injury

**DOI:** 10.7759/cureus.13144

**Published:** 2021-02-05

**Authors:** Javier A Toledo, Rafael Namias, Maria Julia Milano

**Affiliations:** 1 Neurosurgery, Clemente Alvarez Hospital, Rosario, ARG; 2 Neurosurgery Department, Sanatorio Parque - Grupo Oroño, Rosario, ARG; 3 Algorithm Research Department, Brainomix, Oxford, GBR; 4 Radcliffe Department of Medicine, University of Oxford, Oxford, GBR

**Keywords:** traumatic brain injury, ct, automatic analysis, critical care

## Abstract

Introduction

To predict patient outcomes in traumatic brain injury (TBI) lesions, various scores have been proposed, which use objective assessments. These scores, however, rely on the observer's ability to determine them. This study presents a comprehensive, reproducible, and more anatomically stratified objective measurement of the degree of basal cistern effacement in brain computed tomographic (CT) scan images.

Methods

Patients with TBI admitted from August 2015 to February 2016 were included. The control group consisted of non-trauma patients, who had normal brain CT scans. The images were analyzed by an automated volumetric compression ratio (CR) defined as the volume ratio between the parenchymal tissue and the cerebrospinal fluid (CSF) in the basal cisterns. This value was compared with the TBI severity recorded at each patient's admission and a consensus score of the basal cisterns' degree of effacement by manual analysis.

Results

Seventy-three TBI patients were admitted. The mean admission Glasow Coma Scale (GCS) score was 9. In the non-TBI control group, 29 patients were enrolled. The average kappa value for the inter-observer agreement was 0.583. The CR had an inverse linear relationship with the severity of the TBI and the degree of effacement of the basal cisterns. The correlation between the CR value in the midbrain and the specialists' consensus determination was statistically significant (p < 0.01). The CR also showed a difference between the TBI and the control groups (p 0.0001).

Conclusions

The automated CR is a useful objective variable to determine the degree of basal cistern effacement. The proposed ratio has a good correlation with the classical basal cistern effacement classification and TBI severity.

## Introduction

Traumatic brain injury (TBI) lesions are well-characterized on computed tomography (CT) imaging. Different scores, such as the Marshall CT classification, Rotterdam CT score, Helsinki CT scoring system, etc., have been proposed to perform objective assessments of the CT scan-derived metrics used to predict TBI patient outcomes [1-2]. These scores are not accurate, as they rely on the ability of the observer to calculate them. Objectivity is essential when determining the degree of brain swelling. The most relevant brain CT scan imaging characteristics to determine TBI patient outcomes are the midline shift, compression of the basal cisterns at the midbrain level and location of the intracranial haemorrhage (extradural, subdural, etc.), as they are strongly correlated with increased intracranial pressure (ICP) and death [1]. In TBI, the degree of effacement of the basal cisterns is also an important element that can be used to predict a patient's outcome [3], thus it is important that accurate measurements of the latter be performed without inter-observer variability [4-5].

The Brain Trauma Foundation initially proposed a standardized protocol that has become the clinical guideline for increasing interobserver agreement when evaluating basal cistern status [6]. According to the guidelines, mass effect is measured at the midbrain level through a simplified classification of the basal cistern as being completely effaced, partially effaced, or normal. Although this approach has been useful so far, some issues need to be addressed. For instance, different observer interpretations of CT scan images may vary [4]. Also, despite the protocoled acquisition of CT scan slices, the images may not match these standards and may lead to errors in CT scan image interpretations when faced with patient anatomical variations, aberrant gantry inclination, beam hardening, etc. Other research groups have developed tools to assist physicians in evaluating the basal cisterns as well [7]. For example, Yuh et al. proposed an algorithm that measures the volume of cerebrospinal fluid (CSF) within the basal cisterns and determines their status using the standard basal cistern compression classification of the Brain Trauma Foundation described above. The CSF volume measurement within the complete basal cisterns, however, can lead to underestimation of the effacement.

Given the current knowledge gap, this study proposes a powerful, comparable, and more anatomically stratified objective measurement of the effacement of the basal cisterns using a novel compression ratio (CR) within a standard Montreal Neurological Institute (MNI) space. The latter MNI defined a new standard brain by utilizing a large number of CT scans done on normal controls.

## Materials and methods

Patients admitted to Hospital Clemente Alvarez in Rosario, Argentina, from August 2015 to February 2016 were included in the study. The Institutional Research Ethics Committee approved the study protocol.

Patients

Cases assessed in this study were patients admitted because of a TBI, were between 18 and 65 years old, and had their initial brain CT scan performed at our hospital within 12 hours of a TBI injury. Patients with previously known brain abnormalities were excluded. Patients in the control group were admitted because of a non-TBI and had a normal brain CT scan as assessed by a board-certified neuroradiologist.

A case report form was developed, and the patient's data were extracted from the medical records. The variables recorded were age, sex, Glasgow Coma Scale (GCS) score, and the mechanism and kinetics of the trauma resulting in the TBI. 

Imaging protocol

Brain CT scan images were retrieved from the central archive system for both groups consisting of 5 mm-thick contiguous slices acquired with a Toshiba Aquilion 16-slice CT scanner (Tokyo, Japan). All brain CT scan lesions were included with the exception of postoperative images. 

Ground truth

The ground truth consisted of a consensus score of the manual analysis done by two board-certified neurosurgeons and a board-certified neuroradiologist. They independently classified all basal cistern compression/effacement according to the standard Brain Trauma Foundation Classification. Afterward, a consensus between their analyses was reached. The TBI severity, which was classified as mild (GCS score of 14 to 15), moderate (9 to 13), and severe (3 to 8) was considered as well.

Basal compression ratio

A novel feature was proposed to characterize the compression status of the basal cisterns from CT scan acquisitions. The CR value was defined as the volume ratio between the basal cistern CSF and the parenchymal tissue (white matter (WM) and grey matter (GM)) in a specific basal cistern region.

Pre-processing

The first stage consisted of using a standard skull-stripping technique for CT scan acquisitions to isolate brain tissue. The brain tissue was then registered to the MNI space using an affine transformation employing the Advanced Normalization Tools (ANTs) software-provided ants affine script [8].

Registration quality

In the MNI space, each case's segmented parenchyma was compared with the template parenchyma region using an overlap measurement known as the Jaccard Index: J = #(A∩B) / #(A∪B) where J = the Jaccard distance, A = set 1 and B = set 2. The index was 1 when the overlap was complete and 0 when there was no overlap. With this method, the skull-stripping and registration quality were assessed simultaneously for the groups with and without trauma. 

Segmentation and analysis

**Figure 1:**
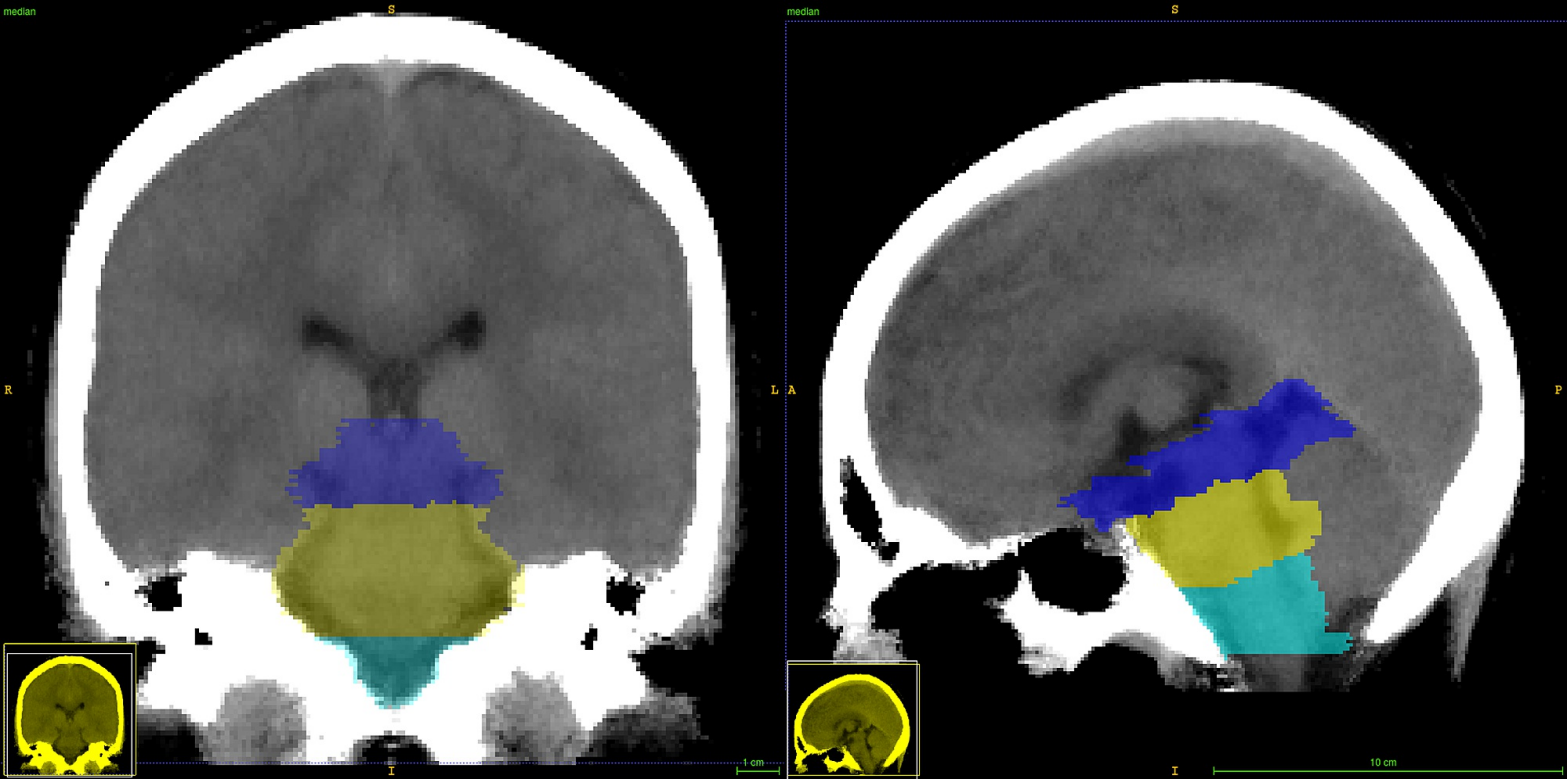
CT template Sagittal and coronal views of the CT template along with the three basal cistern section segmentations: mid-brain (blue) from the superior edge of the quadrigeminal plate to the superior pontine notch, pons (yellow) from the midbrain to the inferior pontine notch, and medulla from the inferior limit of the pons to the superior edge of C1 (turquoise) CT: computed tomography

Within the MNI space template, a manual basal cistern mask was created considering three different parts of this region, i.e., the midbrain, pons, and medulla, as shown in Figure 1. For this purpose, the midbrain's cranial border was anatomically defined as the axial plane through the mammillary body and the superior edge of the quadrigeminal plate. The caudal border was defined by the axial plane aligned to the superior pontine notch and the inferior edge of the quadrigeminal plate. It was determined that the inferior boundary of the pons was composed of a plane parallel to the caudal border plane and aligned with the low pontine notch. The medulla region was defined from the pons' inferior boundary to the superior edge of the first cervical vertebra [9].

Once a scan had been aligned into the MNI space, the basal cistern mask was applied. As a result, a set of voxels of interest within the basal cistern regions was gathered. These voxels were then pre-filtered using a threshold method to remove voxels with intensities lower than -5 Hounsfield units (HUs) or greater than 45 HUs. The remaining set was classified into CSF, GM, or WM using a k-means classic clustering method with three clusters based on intensity only. The clusters were initialized at fixed typical intensities of 0, 25, and 35 HUs representing CSF, GM, and WM, respectively, similar to the method presented by Zhu et al. [10]. Finally, the CR was computed by dividing the number of CSF voxels by the parenchymal tissue voxels (GM + WM). This ratio's values ranged between 0 and a number lower than 1; the basal cisterns were fully effaced when the CR trended to 0. The CR value trended to its natural CSF proportion within the brain stem when the amount of CSF surrounding the brain stem was more significant.

Statistical analysis

The statistical analysis was performed using the IBM Statistical Package for the Social Sciences (SPSS) statistics V.20 software (IBM Corp., Armonk, NY) [11]. Continuous variables were summarized with the mean, minimum, and maximum values and the standard deviation (SD) or median (Q1-Q3 quartile range). To determine differences between the groups, the Mann-Whitney U-test was used to compare the CR medians of the different brain sections against the trauma severity. For the manual basal cistern compression/effacement annotations, an inter-rater variability analysis was performed. Interobserver agreement was determined using Cohen's kappa statistics for qualitative features. The Kruskal Wallis test was used to determine CR differences between the human observer classifications. A Spearman's rho test was utilized to calculate the correlation between groups. A p-value of less than 0.05 was considered statistically significant.

## Results

Patient admission characteristics

From August 2015 to February 2016, 73 patients were admitted with the diagnosis of TBI. The patients' mean age was 36 (+/- 15) years, ranging between 18 and 65. Sixty-one patients were men and 12 were women. The mean admission GCS score was 9, with a range between 3 and 15. Motor vehicle accidents caused TBI in 57 cases, a fall in five cases, violence in two, and in nine instances, the etiology was unknown. In the control group, 29 patients were enrolled; 16 were men and 13 were women. The mean age was 37 (+/- 14) years, ranging between 18 and 52 years (Table 1).

**Table 1:** Admission characteristics of patients enrolled in the study TBI = traumatic brain injury; N = number of patients'; SD = standard deviation; M/F = male/female; GCS = Glasgow Coma Scale; M. = Mechanical

Admission Characteristics			TBI group	Control group
N			73	29
Age (SD)			36 (±15)	37 (±14)
Gender (M/F)			61/12	16/13
TBI Cause				
	Motor Vehicle Accident		57 (79%)	
	Fall		4 (6%)	
	Violence		2 (2%)	
	Unknown		9 (13%)	
Pupils				
	Isocoric		58 (79%)	
	Anisocoric		7 (10%)	
	Bilateral Mydriasis		1 (1%)	
	Unknown		7 (10%)	
GCS Score				
	3-8		38 (52%)	
	9-13		17 (23%)	
	14-15		18 (24%)	
Sedation (%)			55%	
M. Ventilation (%)			58%	
Hypoxia (%)			4%	
Hypotension (%)			3%	
Drugs to Control Edema (%)			5%	
Hemodynamic Instability (%)			38%	
Craniotomy (%)			29%	

Imaging characteristics

The Marshall TBI classification was used as follows: patients who were type I had no visible intracranial pathology; II had a midline shift of 0 to 5 mm, the basal cisterns were visible, and there were no high or mixed density lesions > 25 cm^3^; III had a midline shift of 0 to 5 mm, the basal cisterns were compressed or completely effaced, and there were no high or mixed density lesions > 25 cm^3^; IV had a midline shift > 5 mm with no high or mixed density lesions > 25 cm^3^; V had any lesions evacuated surgically and these patients were excluded from this study; VI had no non-evacuated mass lesion(s) and had high or mixed density lesions > 25 cm^3^ [2]. Most brain CT scan images had diffuse injuries types I and II (Table 2).

**Table 2:** Marshall score classification of the CT images. All the grades were represented in the sample except for grade V (postoperative). TBI = traumatic brain injury; N = number of patients; CR = compression ratio CT = computed tomography

Marshall TBI Classification	N	%	Mean CR
I	23	31%	0,199
II	27	37%	0,179
III	9	12%	0,097
IV	6	8%	0,12
V	0	0%	-
VI	8	11%	0,111

Inter-observer variability

The first analysis assessed the inter-observer agreement when scoring basal cistern effacement. The average inter-observer agreement between all readers was 0.583 (Cohen's kappa coefficient), indicating moderate agreement (Table 3). The consensus value was obtained from the classification of the basal cisterns done by the three observers. The most frequent grading of each case was considered the ground truth. 

**Table 3:** Inter-observer variability (Cohen’s kappa coefficients) between independent observers evaluating basal cistern effacement

Observers	Kappa	p
1	0.296	.000
2	0.611	.000
3	0.842	.000
Average	0.583	.000

Group compression ratio assessment

A comparison of the CR for both groups (the TBI group and control group) was done for the different sections of the brain stem. The midbrain CR medians were 0.242 (0.195-0.260) for the control group and 0.146 (0.063-0.191) for the TBI group; the values for the pons were 0.211 (0.179-0.245) for the control group and 0.146 (0.090-0.190) for the TBI group. The median CRs for the medulla were 0.350 (0.264-0.412) and 0.253 (0.173-0.303) for the control and TBI groups, respectively. For the complete brain stem cisterns, these values were 0.242 (0.195-0.276) for the controls and 0.164 (0.106-0.212) for the TBI group, respectively (Table 4).

**Table 4:** A comparison between the TBI group and the control group compression rates in the various basal cistern sections. Q = quartile; TBI = traumatic brain injury

Brain Stem Segments	Group	Median (Q1-Q3)	
Midbrain	TBI	0.146 (0.074-0.197)	p < 0.0001
	Control	0.242 (0.195-0.261)	
Pons	TBI	0.146 (0.089-0.190)	p < 0.0001
	Control	0.211 (0.167-0.247)	
Medulla	TBI	0.253 (0.167-0.308)	p < 0.002
	Control	0.350 (0.253-0.423)	
Total	TBI	0.164 (0.095-0.214)	p < 0.0001
	Control	0.242 (0.192-0.276)	

Compression ratios for each group's brain stem sections

**Figure 2:**
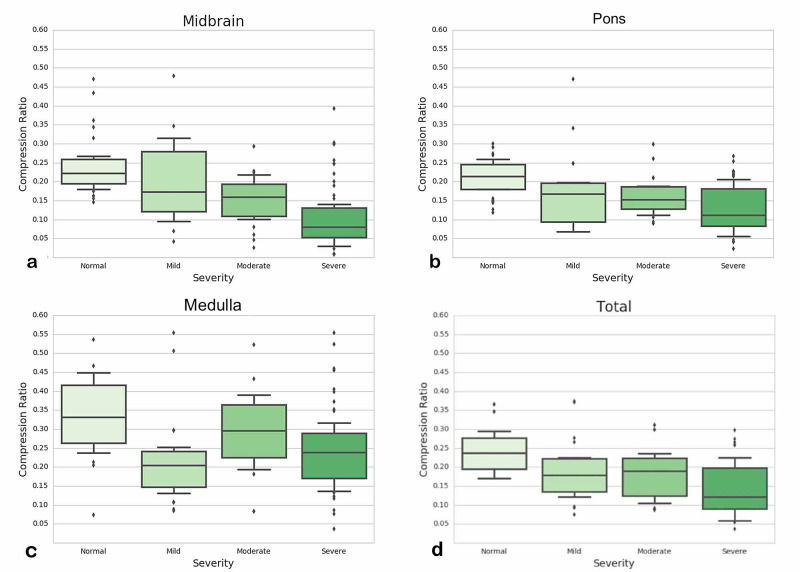
Compression ratio distribution according to the trauma severity in the various basal cistern sections (a) Midbrain, (b) Pons, (c) Medulla, (d) a final analysis taking into account the overall volume of the three sectors

A comparison of the CR values in each brain stem section was performed using a Mann-Whitney's test for the TBI versus the control groups. A statistically significant difference was found between the TBI group and the same sector in the control group (Table 5). A global correlation was found for the CR value distributions grouped by manual annotation of the cases for every brain stem sector. The control group's CR value medians were the largest; they tended to decrease as the severity of the injury increased (Figure 2, from left to right). The midbrain section showed the best linear correlation (Figure 2a).

**Table 5:** Compression ratio variation according to trauma severity in the different cistern sections and total volume TBI = traumatic brain injury; N = number of patients'; CR = compression ratio; SD = standard deviation

Brainstem segments	TBI Severity/Control	N	CR Median (SD)	
Midbrain	Control	29	0.242 (±0.078)	p < 0:0001
	Mild	20	0.204 (±0.105)	
	Moderate	14	0.143 (±0.078)	
	Severe	39	0.117 (±0.087)	
Pons	Control	29	0.211 (±0.049)	p < 0:0001
	Mild	20	0.157 (±0.093)	
			Moderate		14	0.161 (±0.059)
	Severe	39	0.135 (±0.075)	
	Medulla	Control	29		0.350 (±0.141)	p < 0:002
Mild				20	0.232 (±0.157)	
Moderate		14	0.282 (±0.135)			
Severe		39	0.253 (±0.107)			
Total		Control	29	0.242 (±0.051)	p < 0:0001	
	Mild	20	0.186 (±0.080)		
	Moderate	14	0.176 (±0.072)		
	Severe	39	0.148 (±0.074)		

Manual versus automated basal cistern compression grading

**Figure 3:**
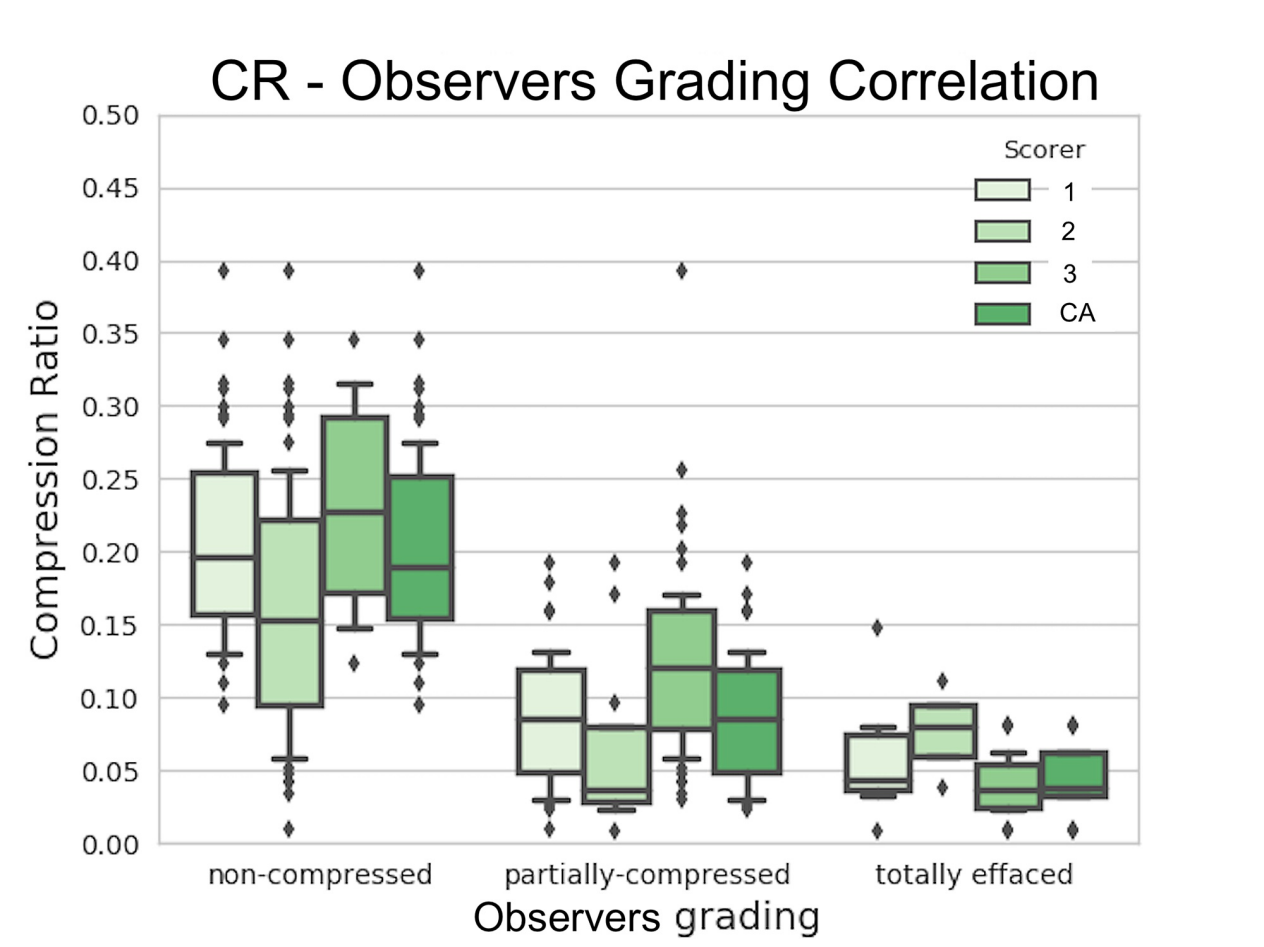
Compression ratio grading Distribution of the midbrain CRs for the manually annotated basal cistern compression grading of the three experts and the consensus annotation. A positive correlation was found between these variables (Spearman’s rho test p<0.05). CR = compression ratio, CA = consensus annotation

The median of the CR values for non-compressed basal cisterns was 0.215 (0.164-0.260), for the partially-compressed basal cisterns, 0.090 (0.049-0119), and for the effaced basal cistern group, 0.047 (0.034-0.072) (Table 6). For the consensus of the basal effacement categories, the Kruskal-Wallis test was used to assess the similarity of CR values. A significant similarity was found between all groups except for the medulla (p=0.457). A positive correlation was found between the midbrain's CR values and the specialist's consensus for the standard basal cistern compression classification (p<0.05). Figure 3 shows the distribution of the CR values for the midbrain versus the consensus grading annotation. A decreasing correlation was found between the Brain Trauma Foundation basal cistern effacement grading and the CR median values in all sections. It was not possible, however, to establish a strong linear relationship between the CR value and the physicians' measurement for medulla effacement (Table 7).

**Table 6:** Degree of compression variation according to trauma severity in the different cistern sections and the total represented by the median and the Q1-Q3 interval Q = quartile

Degree of compression	Midbrain (Q1-Q3)	Pons (Q1-Q3)	Medulla (Q1-Q3)	Total (Q1-Q3)
Non-compressed	0.215 (0.154-0.270)	0.190 0(0.149 - 0.212)	0.299 (0.216 - 0.367)	0.218 (0.178 - 0.263)
Partially compressed	0.090 (0.049-0119)	0.110 (0.826 - 0.119)	0.215 (0.148 - 0.280)	0.120 (0.915 - 0.125)
Totally effaced	0.047 (0.033-0.075)	0.083 (0.041 - 0.097 )	0.184 (0.133 - 0.252)	0.087 (0.071 - 0.09)

**Table 7:** Correlation between human measure classification and compression ratio Note: Correlation is significant at the 0.01 level (2-tailed). Sig. = Significance; N = number of patients

Spearman's rho	Midbrain	Pons	Medulla	Total	Consensus
Correlation Coefficient	0.789^**^	0.651^**^	0.377^**^	0.766^**^	1.000
Sig. (2-tailed)	.000	.000	.001	.000	.
N	73	73	73	73	73

## Discussion

In this study, a novel technique is presented that objectively characterizes basal cistern compression in human brains. This technique has several advantages. The images are analyzed volumetrically in an aligned space; this compensates for the discrepancies that may appear in CT scan acquisitions. Also, the analysis is both objective and reproducible since the overall method is entirely automated. The CR proved to have a reasonable correlation with the manual classification for basal cistern compression and effacement.

A key issue with the interpretation of CT scan images is the inter-observer variability. Several scores have been proposed to assess significant clinical features and to standardize the observations. The most widely used is the Marshall score, as it is simple and able to predict prognosis in TBI. Although this classification is quite pragmatic, it has limitations such as difficulties in the delineation of patients with concomitant types of brain injuries and in the standardization of certain features of brain CT scan images.

The Rotterdam score has made the CT scan imaging evaluation more straightforward. It has not been fully validated, however, and requires the imaging to be studied in depth [9]. Regardless of the classification used, there are other limitations with CT scan acquisition techniques and the human evaluators' ability to make a volumetric analysis. For instance, different gantry or cranial tilt inclinations may lead to overestimation of the basal cistern compression or midline shift. These discrepancies may result in inaccurate outcome prognoses of the patients. The developed tool compensates for this issue to some extent by straightening the image and presenting a realigned reconstruction of it. This provides the reader with a more standard framework with which to assess the scan.

Specialists evaluate the degree of basal cistern compression at the midbrain cistern level only. It was thus reasonable to compare the manual effacement with the median CR values for the midbrain section.

Another similar analysis tool was described by Yuh et al. [7]. This work had a classical qualitative classification approach by automatically assigning the scans the TBI severity grade of mild, moderate, or severe. The method used in the present study gives a novel quantitative parameter that opens the field to further validation of this variable to predict the outcome in TBI. Even though it was not possible to compare the CR feature against the tool used by Yuh et al., our method certainly provides a more accurate anatomical stratification of the basal cistern sections. The midbrain section correlated the most with the specialist grading scores; its analysis could constitute a more accurate indicator of the degree of basal cistern compression.

The quality of the automated CR value has proved to be very powerful, even with the presence of hemorrhages, midline shift, and other image abnormalities present in TBI patients. The clustering results for the tissue characterization stage were visually validated since the manual annotation was cumbersome and time-consuming.

The CR values have an inverse linear correlation with the severity of the TBI and the basal cisterns' effacement. This inverse relationship is preserved in all but the medulla section of the basal cisterns. Thus, the CR value is straightforward to understand. It has also proved to be useful for brain CT scan imaging assessment. This feature, however, is only one of the other vital characteristics in brain CT scan images that correlate with the patients' TBI severity and functional outcome.

In the future, the expansion of the presented methodology could automatically evaluate most of the relevant brain CT scan imaging features for TBI patients such as midline shift and hemorrhage volume. Combining clinical variables with the automated feature extraction in brain CT scan images could predict short-term clinical evolution and provide support to make clinical decisions. Further studies are necessary to evaluate its application in clinical practice to predict neurological worsening and functional outcomes.

## Conclusions

The automated CR demonstrated that it is a useful objective variable to determine basal cistern effacement. The proposed ratio has a good correlation with the classical qualitative basal cistern effacement classification and TBI severity.
